# Exploring core and sentinel symptoms in elderly patients with type 2 diabetes through network analysis and the Apriori algorithm

**DOI:** 10.3389/fmed.2026.1728665

**Published:** 2026-03-11

**Authors:** Tingting Tan, Chongyao Yang, Qingqing Wang, Cong Xu, Sijing Wang, Bingbing Xiao, Jing Zhao, Huan He, Miaoqin Wang

**Affiliations:** 1 Department of nursing, Affiliated Hospital of North Sichuan Medical College, Nanchong, China; 2 North Sichuan Medical College, Nanchong, China; 3 West China School of Public Health and West China Fourth Hospital, Sichuan University, Chengdu, China; 4 Department of Stomatology, Affiliated Hospital of North Sichuan Medical College, Nanchong, China; 5 Outpatient Department, Affiliated Hospital of North Sichuan Medical College, Nanchong, China

**Keywords:** elderly patients with type 2 diabetes mellitus, sentinel symptoms, core symptoms, network analysis, symptom management

## Abstract

**Objective:**

To construct the symptom network of elderly patients with type 2 diabetes mellitus (T2DM) and perform association rule analysis for each symptom cluster, identify core symptoms, and explore the sentinel symptoms, to provide references for accurate and efficient symptom management.

**Methods:**

Convenience sampling was employed. Eligible elderly patients with T2DM were enrolled from the department of endocrinology, affiliated hospital of North Sichuan Medical College, Nanchong City, from March 2024 to July 2024, with data collected using the Chinese Version of Diabetes Symptom Checklist-Revised (DSC-R). The correlation was analyzed using the Apriori algorithm model to identify the sentinel symptoms of each symptom cluster, and the core symptom was identified based on the network analysis method.

**Results:**

The most prevalent symptoms were thirst and dry mouth (71.69%), general fatigue (70.78%), and frothy urine (69.58%). Regarding symptom burden severity, general fatigue (2.48 ± 1.89), asthenia (2.42 ± 1.94), and insomnia (2.39 ± 1.98) had the highest scores. Sentinel symptoms, including excessive sleepiness, limb numbness, limb swelling, and polydipsia, are characteristic manifestations of distinct symptom clusters: psychological cognition symptom cluster, peripheral nerve symptom cluster, nephropathy symptom cluster, and hyperglycemia symptom cluster. At the global level, general fatigue (r_s_ = 1.207) emerged as the core symptom.

**Conclusion:**

This study identifies general fatigue as the core symptom, with excessive sleepiness, limb numbness, limb swelling, and polydipsia emerging as sentinel symptoms, within their respective symptom clusters. From the perspective of symptom network analysis and sentinel symptoms, clinical staff can develop accurate and efficient intervention measures to improve the efficiency of symptom management and reduce the burden of symptoms in elderly patients with T2DM.

## 1 Introduction

Diabetes mellitus (DM) is a chronic metabolic disorder characterized by persistent hyperglycemia due to impaired insulin secretion or defective insulin action, and is currently classified by the World Health Organization (WHO) as one of the four priority non-communicable diseases (1). According to the data from the International Diabetes Federation (IDF) (2), approximately 11.1% of the global population aged 20–79 years (589 million people) are living with DM as of 2024. The IDF Diabetes Atlas reports that China has the highest number of cases worldwide, with approximately 140 million patients, accounting for one-quarter of the global total (3). Type 2 diabetes mellitus (T2DM) represents the majority of cases, comprising 90%−95% of all DM diagnoses (4). It is defined by insulin resistance—a diminished biological response to insulin—coupled with a relative, rather than absolute, deficiency in insulin secretion (4). Elderly diabetes refers to diabetes in individuals aged ≥ 60 years (or ≥65 years according to WHO criteria), comprising patients diagnosed before and after this threshold (1). Due to prolonged disease progression, elderly patients with T2DM frequently present with multiple concurrent symptoms, including fatigue, pain, depression, sleep disturbances, and emotional dysfunction (5, 6). These symptoms typically manifest in a complex, interrelated manner, often forming distinct symptom clusters. Symptom clusters are defined as a group of two or more interrelated symptoms that occur simultaneously (7). Symptoms within a cluster exhibit dynamic interrelationships characterized by mutual reinforcement, creating a self-perpetuating cycle that exacerbates disease complications and diminishes patients' quality of life (8). Current research on T2DM has predominantly focused on individual symptoms or overall symptom burden. However, a systematic investigation of the intricate interactions between co-occurring symptoms is lacking (5, 6, 9). Consequently, current symptom management strategies are inefficient, resulting in resource wastage and increased healthcare costs. Therefore, identifying key intervention points requires a dual focus: the underlying mechanisms within symptom clusters and global symptom patterns. Specifically, at the cluster level, it is necessary to determine whether a specific symptom can predict other symptoms within the same cluster. Defined as early warning indicators, sentinel symptoms are identified via the Apriori algorithm as probabilistic antecedents that predict the onset of related symptoms within a cluster—even when derived from cross-sectional data—thereby providing a statistical basis for the clinical prevention of symptomatic escalation (10, 11). At the global level, it is necessary to explore which symptoms act as ^**^key triggers^**^ for other symptoms.

In recent years, symptom network analysis has gradually emerged as a research focus in symptom science. Symptom network models can characterize the overall interactive relationships among different symptoms. This approach enables the identification of core symptoms that are most likely to increase the risk of other symptoms (12), suggesting that optimal management strategies can be developed by leveraging the synergistic relationships within symptom clusters. In conclusion, sentinel, core, and high-prevalence symptoms play distinct clinical and structural roles within symptom clusters. Sentinel symptoms act as warning indicators that precede the evolution of symptom clusters, providing critical early-warning value for adverse clinical outcomes (11). In contrast, core symptoms are characterized by their structural centrality in symptom networks; as high-centrality nodes, they exert a dominant influence on overall network structure and the expression of peripheral symptoms (13). The research value of sentinel symptoms lies in identifying intervention windows, while the value of core symptoms is to dismantle the symptom system. High-prevalence symptoms, while reflecting common disease manifestations, do not directly indicate the risk of adverse clinical outcomes.

The integration of Apriori analysis and symptom network modeling provides a robust framework for investigating symptom mechanisms at both cluster-specific and systemic levels. It is therefore hypothesized that sentinel symptoms play a crucial role in triggering the onset of other symptoms within a cluster. Individual symptoms and symptom clusters interconnect with one another, ultimately forming a comprehensive symptom network. Within this network, sentinel and core symptoms act as key indicators of symptom occurrence and progression at both the cluster and global levels. These symptoms represent strategic targets for interventions designed to disrupt pathological symptom associations across multiple organizational levels, thereby optimizing the efficacy of symptom management strategies (8, 14). Despite their complementary nature, sentinel symptom analysis and symptom network modeling have largely been applied in isolation, resulting in a fragmented understanding of symptom pathogenesis (15, 16). While a study has been conducted to construct symptom networks in patients with T2DM, a relevant study by Liu et al. presented some differences: the study cohort did not focus on the specific population of elderly patients with T2DM, and only network analysis was used to explore core symptoms, with no in-depth study on the marker symptoms associated with the progression of symptom clusters (17). This study seeks to dismantle the symptom system by identifying core symptoms and pinpoint intervention windows by detecting sentinel symptoms within symptom clusters—an approach that can better assist clinical practitioners in managing T2DM-related symptoms and improve the efficiency of symptom management. Therefore, the present study aimed to integrate sentinel symptom analysis and symptom network modeling to identify (1) sentinel symptoms within individual clusters and (2) core symptoms of the overall symptom network.

## 2 Materials and methods

### 2.1 Study design and sample

A cross-sectional study was conducted with a convenience sample of 332 elderly patients with T2DM. Participants were recruited from the department of endocrinology at the affiliated hospital of North Sichuan Medical College in Nanchong. Recruitment took place from March 2024 to July 2024. This exploratory study aimed to identify sentinel symptoms and symptom association patterns in elderly patients with T2DM. The convenience sampling method allowed for efficient recruitment and provided an adequate sample size. This supported the use of association rule mining and symptom network analysis. The approach also leveraged existing clinical resources. It helped streamline participant screening and data collection. Furthermore, it supported control over the study timeline and budget.

Inclusion Criteria were as follows: (1) Age ≥ 60 years; (2) diagnosis of T2DM confirmed by clinical physicians; (3) ability to communicate clearly; (3) voluntary participation with written informed consent. Exclusion Criteria were as follows: (1) Severe mental illness or cognitive impairment; (2) other major somatic diseases; (3) participation in other similar studies.

A questionnaire comprising 34 symptoms was employed in this study. To ensure symptom network stability, according to previous studies and clinical experience (18, 19), symptoms with an incidence of less than 25% could be excluded, including chest tightness, diarrhea, palpitations, nausea, and vomiting, abdominal distension, burning sensation in the lower extremities, shortness of breath, hand tremor, chest pain, decreased appetite, cold sweats, could be excluded, resulting in 23 retained symptoms.

The minimum sample size for network analysis was calculated using the formula P (P-1)/2, where P denotes the number of symptom nodes. For 23 symptoms, the minimum required sample size was 253. In this study, 332 participants were included, exceeding this threshold. Of the 340 questionnaires distributed, 332 valid responses were collected, yielding a response rate of 97.6%. This cross-sectional study involving human participants was approved by the Ethics Committee of the Affiliated Hospital of North Sichuan Medical College (2024ER116-1).

### 2.2 Research tools

#### 2.2.1 Demographic and clinical characteristics

In this study, comprehensive baseline data were collected, including sociodemographic variables (age, sex, educational attainment, marital status) and disease—related variables (duration of T2DM, treatment modalities, diabetic complications, diabetes education, and regularity of glucose monitoring).

#### 2.2.2 Chinese version of diabetes symptom checklist-revised (DSC-R)

The symptom severity subscale of the Diabetes Symptom Checklist-Revised (DSC-R) was used to assess the prevalence and severity of symptoms in elderly patients with T2DM. The DSC-R was originally developed by Snoek et al. in 1994 (20) and revised in 2009 (21), and adapted into Chinese by Luo Qian in 2023 (22). The scale is constructed to assess self-reported symptom burden in individuals with T2DM. It consists of 34 items across eight dimensions: psychological cognition symptoms, peripheral nerve symptoms, gastrointestinal symptoms, hypoglycemia symptoms, ocular symptoms, nephropathy symptoms, hyperglycemia symptoms, and cardiovascular symptoms. Severity is rated on a 5-point Likert scale, with a total possible score of 170 and higher scores indicating greater symptom burden. The subscale demonstrated good internal consistency, with a Cronbach's α of 0.920. The reliability and validity of the DSC-R have been established in several countries, including the Netherlands, the United States, South Korea, and Turkey (23–25).

### 2.3 Survey methods

The survey team, comprising the principal investigator and four nursing graduate students, received uniform training prior to data collection. Using standardized instructions, the investigators explained the purpose and procedures of the study to the participants, obtained written informed consent, and administered the questionnaire. For participants who required assistance, the investigators provided guided completion. In total, 340 questionnaires were distributed, of which 332 valid responses were collected, resulting in an effective response rate of 97.6%.

### 2.4 Statistical analyses

First, SPSS version 25.0 was used to analyze symptom incidence and severity. Normally distributed continuous variables were expressed as the mean ± standard deviation, whereas non-normally distributed continuous variables were reported as the median (interquartile range, P_25_-P_75_). Categorical variables were presented as frequencies (%).

Second, sentinel symptoms were identified using IBM SPSS Modeler 18.0 (IBM Corporation, Armonk, NY, United States). The Apriori algorithm was applied to perform association rule mining of symptom clusters for this purpose (26). However, given the cross-sectional design, the identified sentinel symptoms should be interpreted as predictive markers rather than causal precursors. Although the directionality suggested by the Apriori algorithm indicates statistical associations rather than temporal sequences, the clinical relevance of detecting such symptoms lies in offering actionable early-warning indicators. Monitoring the emergence or change of sentinel symptoms enables targeted interventions prior to symptom progression or development of adverse outcomes. This early-warning strategy shifts symptom management from a reactive “treat-after-appearance” approach toward proactive prevention and precise intervention in diabetes care. Over the past decade, association rule mining algorithms have been increasingly used in medical research to uncover latent relationships among clinical variables and phenotypic characteristics (27, 28). The Apriori algorithm, a core method in association rule learning, can identify frequent co-occurring item sets and generate meaningful association rules based on metrics such as support, confidence, and lift (29). Support indicates the overall frequency of co-occurrence of a specific symptom in the entire sample, whereas confidence reflects the conditional probability that a consequent symptom appears given the presence of an antecedent one (29). This unsupervised machine learning approach incorporated two validation metrics: support (the co-occurrence prevalence between sentinel and subsequent symptoms) and confidence (the conditional probability of consequent symptoms given sentinel symptoms, adjusted for baseline frequencies) (30). Referring to previous studies (29–33), the criterion for determining the sentinel symptoms in each symptom cluster was set to support > 40%, confidence > 60%, confidence > support, and lift degree > 1.

Third, the symptom network was constructed using the qgraph package in R 4.4.3 (Epskamp, S., et al., Amsterdam, Netherlands), based on a Gaussian graphical model estimated using the EBICglasso algorithm. Symptoms were represented as nodes, and the connections between them were represented as edges, with edge thickness reflecting the strength of the association. Core symptoms were identified based on three centrality indices: strength (the sum of a symptom's direct connections with other symptoms), closeness (the inverse of the shortest path distances, reflecting a symptom's position in the network), and betweenness (the frequency of a symptom lying on the shortest paths between other symptom pairs, indicating its bridging role) (34). Network stability was rigorously evaluated using the bootnet (Epskamp, S., et al., Amsterdam, Netherlands) package with 1,000 bootstrap samples. The correlation stability coefficient (CS—C) was calculated for edge weights, with thresholds of ≥ 0.25 considered acceptable and ≥ 0.5 optimal (35). Furthermore, 95% confidence intervals for bootstrap edge weights were constructed to quantify the reliability of edge weights in the network.

## 3 Results

### 3.1 Demographic and clinical characteristics

A total of 332 valid questionnaires were collected. The sociodemographic characteristics of the participants were presented in Table 1. The mean age of the patients was 70.73 ± 6.39 years, and the mean duration of T2DM was 12.38 ± 8.37 years. Most participants were female (*n* = 174, 52.41%), had a primary school education level (*n* = 130, 39.16%), and were married (*n* = 271, 81.63%). In terms of disease-related variables, the majority were treated with oral hypoglycemic agents (*n* = 131, 39.46%), had not received formal diabetes education (*n* = 181, 54.52%), had been diagnosed with diabetic complications (n = 179, 53.92%), and did not perform regular blood glucose monitoring (*n* = 178, 53.61%).

**Table 1:** Socio—demographic characteristics of elderly patients with T2DM (*N* = 332).

**Variables**	**M ±SD or *N* (%)**	***P***
**Age (years)**	70.73 ± 6.39	0.595
**Duration of T2DM (years)**	12.38 ± 8.37	< 0.001
**Sex**		0.200
Male	158 (47.59)	0.200
Female	174 (52.41)	
**Educational attainment**		0.002
No formal education	113 (34.04)	
Primary school education	130 (39.16)	
Junior high school education	45 (13.55)	
Senior high school education	30 (9.04)	
Tertiary education or higher	14 (4.22)	
**Marital status**		0.095
Never married	3 (0.90)	
Married	271 (81.63)	
Divorced	44 (13.25)	
Widowed	14 (4.22)	
**Treatment modality**		< 0.001
Untreated	35 (10.54)	
Insulin therapy	103 (31.02)	
Oral hypoglycemic agents	131 (39.46)	
Hypoglycemic agents +insulin therapy	63 (18.98)	
**Diabetes education receipt**		0.755
Yes	151 (45.48)	
No	181 (54.52)	
**Diabetic complication diagnosis**		< 0.001
Yes	179 (53.92)	
No	153 (46.08)	
**Glucose monitoring regularity**		0.503
Yes	154 (46.39)	
No	178 (53.61)	

### 3.2 Results of the study on the prevalence and severity of symptoms in elderly patients with T2DM

The most prevalent symptoms were thirst and dry mouth (71.69%), general fatigue (70.78%), and frothy urine (69.58%). In terms of severity, the highest symptom burden scores were reported for general fatigue (2.48 ± 1.89), asthenia (2.42 ± 1.94), and insomnia (2.39 ± 1.98). These results were presented in Table 2.

**Table 2:** The Prevalence and burden of elderly patients with T2DM related symptoms (*N* = 332).

Symptom clusters		***N* (*n*)**	**Prevalence (%)**	**Severity of symptom burden (M ±SD)**
Psychological cognition	Slowed cognition	219	65.96	2.01 ± 1.66
	asthenia	228	68.67	2.42 ± 1.94
	Excessive sleepiness	192	57.83	1.79 ± 1.76
	Impaired concentration	216	65.06	2.00 ± 1.72
Peripheral nerve	Limb numbness	178	53.61	1.57 ± 1.73
	Limb pain	173	52.11	1.63 ± 1.79
	Abnormal sensation in the leg or foot	95	58.73	1.80 ± 1.76
	Soreness in the calf when walking	195	58.73	1.89 ± 1.81
Gastrointestinal	Insomnia	170	51.2	1.44 ± 1.59
Eye	Visual disturbances (flashes or black spots)	137	41.27	1.06 ± 1.41
	Deterioration of visual acuity	203	61.14	1.06 ± 1.41
	Blurred vision	222	66.87	2.05 ± 1.73
Nephropathy	Limb swelling	142	42.77	1.31 ± 1.70
	Frothy urine	231	69.58	2.04 ± 1.61
	Insomnia	218	65.66	2.39 ± 1.98
	Itchy skin	181	54.52	1.63 ± 1.66
Hyperglycemia	Polydipsia	180	54.22	1.63 ± 1.72
	Polyuria	216	65.06	2.02 ± 1.77
	General fatigue	235	70.78	2.48 ± 1.89
	Thirsty and dry mouth	238	71.69	2.24 ± 1.69
Hypoglycemia	feeling gloomy	187	56.33	1.88 ± 1.85
	Irritable and easily angered	195	58.73	1.96 ± 1.81
	Dizziness	147	44.28	1.22 ± 1.56

### 3.3 Sentinel symptoms of each symptom cluster in elderly patients with T2DM

Using the Apriori algorithm with criteria of support > 40% and confidence > 60% (where confidence > support), association rule analysis identified excessive sleepiness as the sentinel symptom for the psychological-cognition symptom cluster in elderly patients with T2DM. Under the same criteria, limb numbness was identified as the sentinel symptom for the peripheral nerve symptom cluster, limb swelling for the nephropathy symptom cluster, and polydipsia for the hyperglycemia symptom cluster. No clear sentinel symptom was identified for the hypoglycemic symptom cluster, as dizziness did not meet the criteria for inclusion in the association rules. Similarly, no distinct sentinel symptom was identified for the eye symptom cluster. These findings were summarized in Table 3.

**Table 3:** The sentinel symptoms of each symptom cluster based on the Apriori algorithm.

**Symptom clusters**	**Antecedent**	**Consequent**	**Support (%)**	**Confidence (%)**	**Lift**
Psychological cognition	Excessive sleepiness	Impaired concentration	57.831	71.354	1.097
	Excessive sleepiness	Slowed cognition	57.831	71.354	1.082
	Asthenia	Impaired concentration	68.674	74.122	1.139
	Asthenia	Slowed cognition	68.674	74.439	1.144
	Slowed cognition	Impaired concentration	65.963	78.082	1.200
	Impaired concentration	Asthenia	65.060	78.240	1.139
	Slowed cognition	Asthenia	65.963	78.538	1.144
	Impaired concentration	Slowed cognition	65.060	79.166	1.200
	Excessive sleepiness	Asthenia	57.831	80.729	1.175
Peripheral nerve	Limb numbness	Abnormal sensation in the leg or foot	53.614	80.898	1.377
	Limb pain	Soreness in the calf when walking	52.108	79.190	1.348
	Limb pain	Abnormal sensation in the leg or foot	52.108	78.034	1.328
	Abnormal sensation in the leg or soot	Limb numbness	58.734	73.846	1.377
	Limb numbness	Soreness in the calf when walking	53.614	70.786	1.205
	Calf soreness when walking	Limb pain	58.734	70.256	1.348
Hypoglycemia	Feeling gloomy	Irritable and easily angered	56.325	75.401	1.283
	Irritable and easily angered	Feeling gloomy	58.734	72.307	1.283
Eye	Visual disturbances (flashes or black spots)	Blurred vision	41.265	83.941	1.255
	Deterioration of visual acuity	Blurred vision	61.144	81.773	1.222
	Blurred vision	Deterioration of visual acuity	66.867	74.774	1.222
Nephropathy	Limb swelling	Frothy urine	42.771	78.169	1.123
	Limb swelling	Insomnia	42.771	75.352	1.148
	Itchy skin	Frothy urine	54.518	72.376	1.040
	Insomnia	Frothy urine	65.663	71.560	1.028
	Itchy skin	Insomnia	54.518	69.061	1.052
	Frothy urine	Insomnia	69.578	67.532	1.028
	Limb swelling	Itchy skin	42.771	61.972	1.137
Hyperglycemia	Polydipsia	Thirsty and dry mouth	54.216	85.000	1.185
	Polyuria	Thirsty and dry mouth	65.060	80.555	1.124
	General fatigue	Thirsty and dry mouth	70.783	80.000	1.115
	Thirsty and dry mouth	General fatigue	71.686	78.991	1.115
	Polydipsia	Polyuria	54.216	78.333	1.204
	Polydipsia	General fatigue	54.216	77.777	1.098
	Polyuria	General fatigue	65.060	77.777	1.098
	Thirsty and dry mouth	Polyuria	71.686	73.109	1.123
	General fatigue	Polyuria	70.783	71.489	1.098

### 3.4 Network analysis of elderly patients with T2DM symptoms

**Figure 1:**
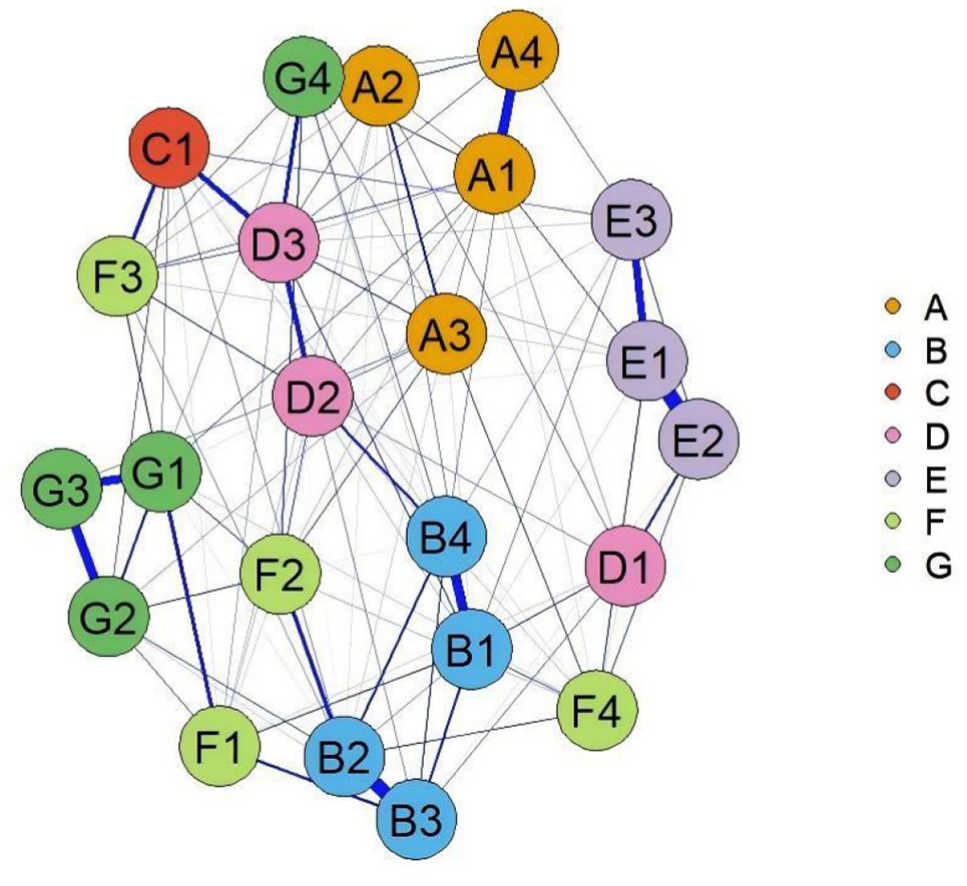
Symptom network of elderly patients with T2DM. For detailed explanations of symptom codes, please refer to Supplementary Table S1.

**Figure 2:**
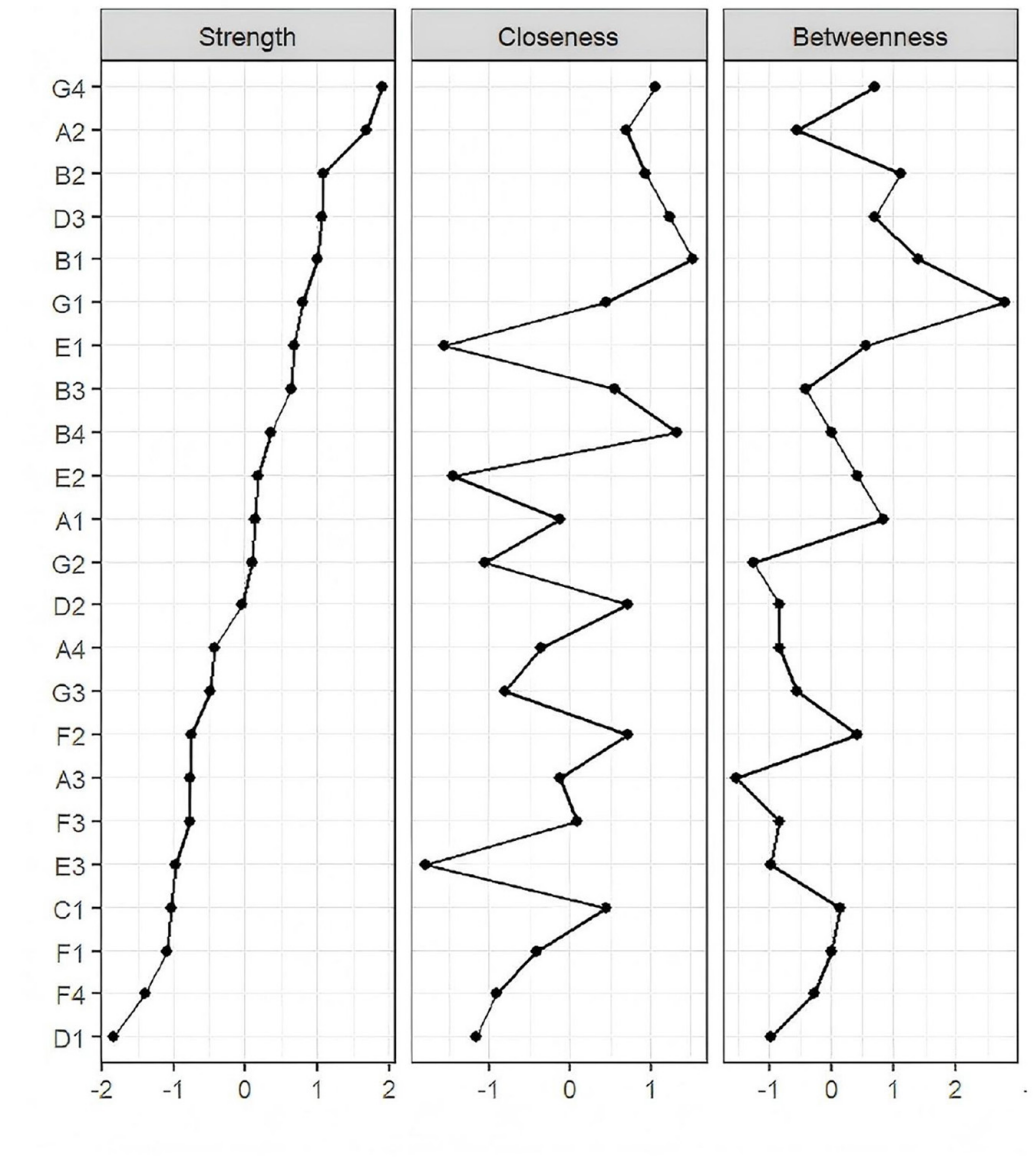
Symptom network node centrality index. Idem.

**Figure 3:**
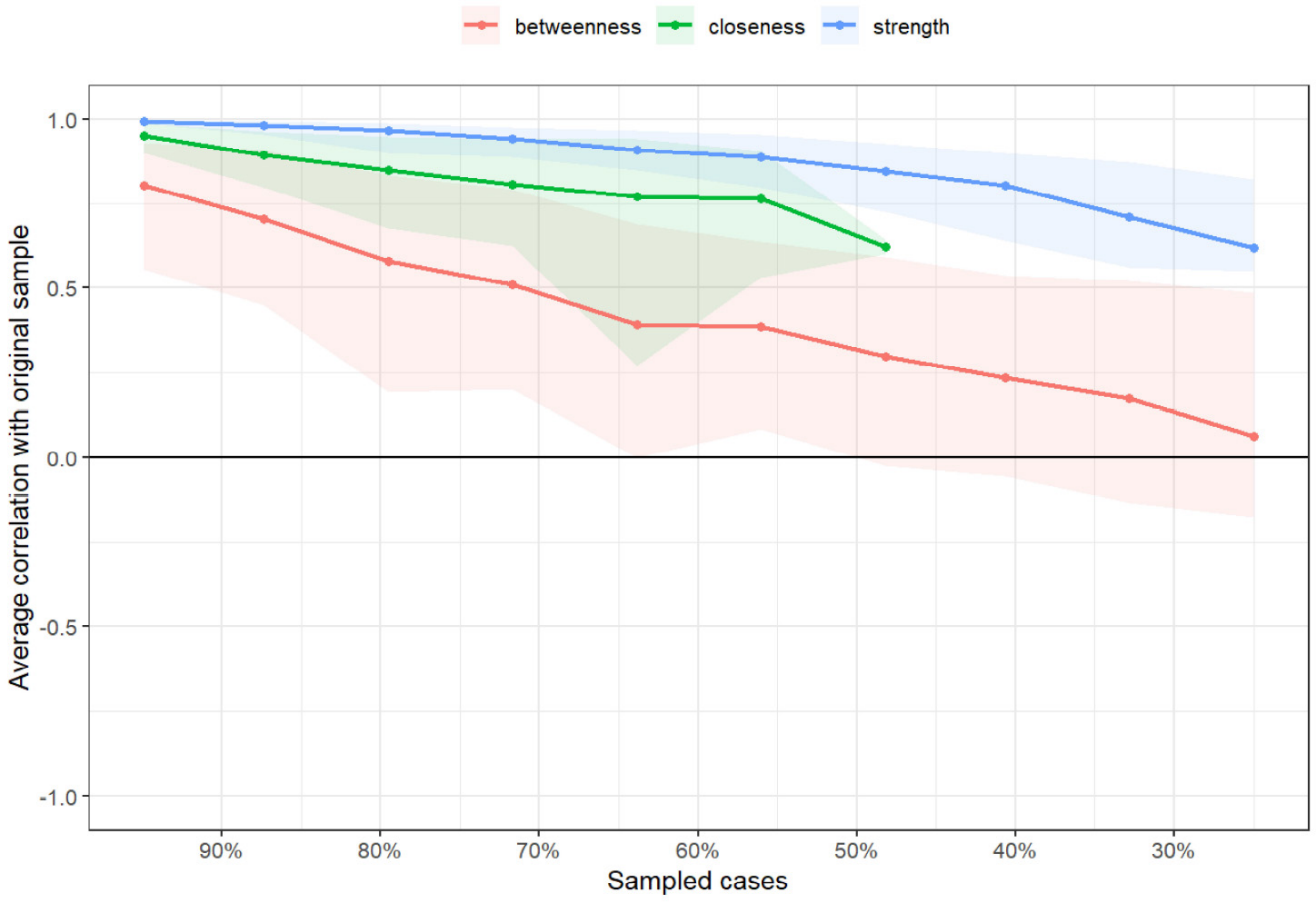
Stability analysis of the symptom network.

**Figure 4:**
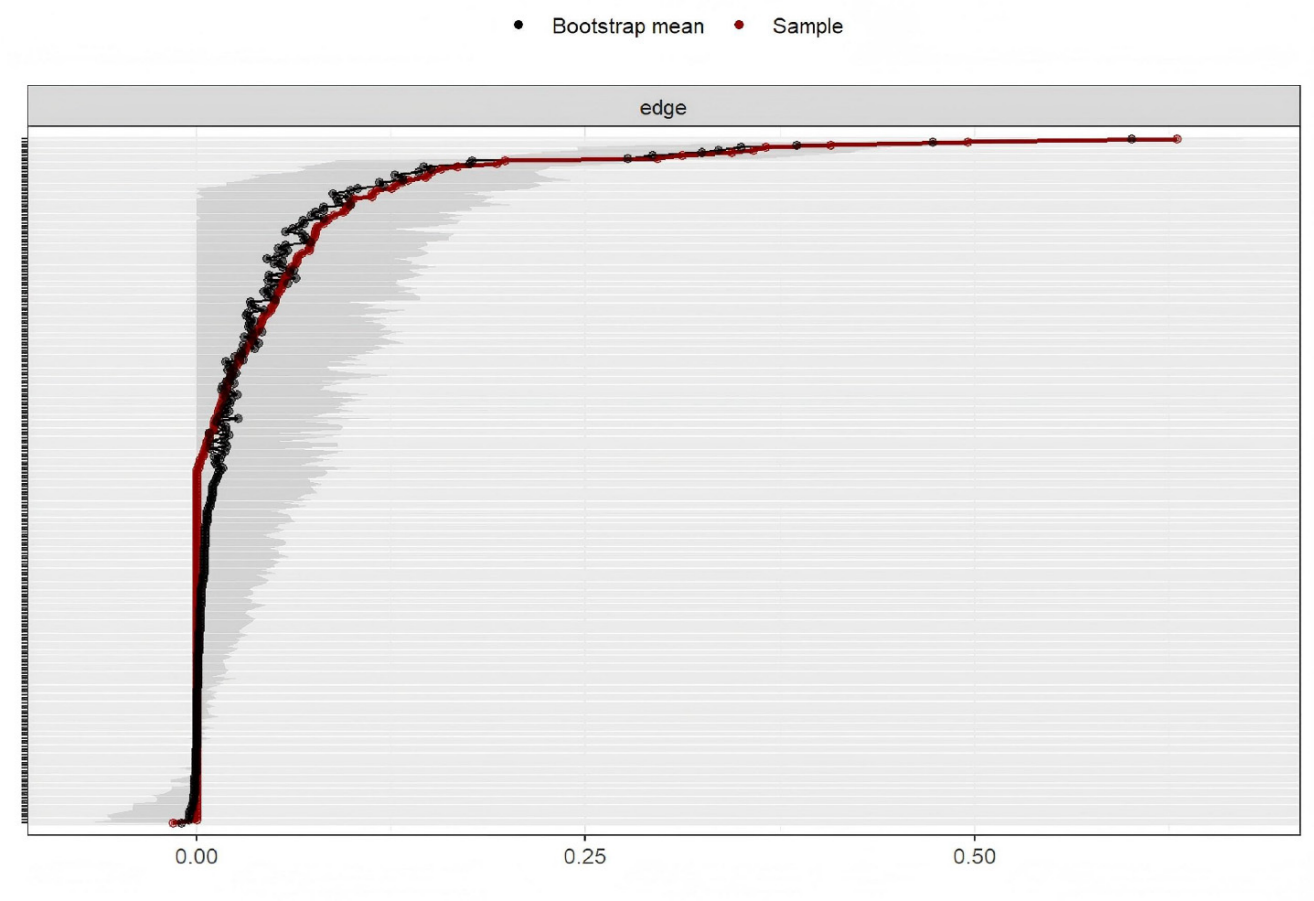
Self-estimation analysis of network edge weights of symptoms.

In the symptom network of elderly patients with T2DM, asthenia had the strongest positive association with general fatigue (Figure 1). Centrality analysis indicated that the three symptoms with the highest strength centrality values were general fatigue (r_s_ = 1.207), asthenia (r_s_ = 1.160), and abnormal sensation in the leg or foot (r_s_ = 1.034). For closeness centrality, the highest-ranking symptoms were soreness in the calf when walking (r_c_ = 0.0026), limb pain (r_c_ = 0.0025), and feeling gloomy (r_c_ = 0.00254). Betweenness centrality analysis identified thirst and dry mouth (r_b_ = 68.00), soreness in the calf when walking (r_b_ = 48.00), and abnormal sensation in the leg or foot (r_b_ = 44.00) as the most central. General fatigue demonstrated the highest strength centrality, confirming its role as the core symptom in this population (Figure 2). The stability analysis of centrality indices showed a stability coefficient of 0.518 for strength centrality (Figure 3). Previous studies demonstrated that closeness and betweenness centrality indicators are unstable (36). Therefore, this study selected strength centrality as the primary criterion for core symptom identification. The edge weight analysis revealed narrow 95% confidence intervals, indicating good accuracy of the network (Figure 4).

## 4 Discussion

To the best of our knowledge, this study represents the first application of integrated network analysis and association rule mining to investigate sentinel symptom and core symptoms in elderly patients with T2DM. This combined analytical approach enables a multilevel examination of symptom interaction patterns, spanning from localized symptom clusters to systemic network manifestations. The proposed model provides a novel conceptual framework for understanding symptom pathogenesis, while concurrently identifying potential targets for clinical intervention. Mechanistically, symptom clusters can be effectively explained through key symptomatic linkages: sentinel symptoms act as early indicators that predict the onset or progression of other symptoms within a cluster, distinguished by their sensitivity, clinical relevance, prevalence, and pathogenic impact (10). The identified sentinel symptoms (excessive sleepiness, limb numbness, limb swelling, and polydipsia) reveal potential mechanisms of symptom aggregation, thereby offering optimal targets for early clinical intervention. Simultaneously, the symptom network model elucidates critical nodes and complex inter-symptom relationships, suggesting that modulating core symptoms can disrupt pathological progression within the network. In practice, priority should be given to the management of sentinel symptoms due to their predictive value for the development of subsequent symptoms, including core ones. Early intervention on sentinel symptoms may generate a cascading inhibitory effect, thereby alleviating the overall symptom burden. This dual—level approach—targeting sentinel symptoms for early warning and core symptoms for network modulation—represents a resource—efficient management strategy, preventing downstream symptom propagation while optimizing therapeutic resource allocation.

### 4.1 Excessive sleepiness has been identified as the sentinel symptom within the psychological cognition symptom cluster

This cluster comprises slowed cognition, asthenia, and impaired concentration, with excessive sleepiness identified as the sentinel symptom. Excessive sleepiness, identified as its sentinel symptom, exhibits a significantly higher prevalence in elderly patients with T2DM compared to the general population (5, 37, 38). The pathophysiological mechanisms linking excessive sleepiness to other symptoms within this cluster are likely multifactorial. Potential contributors may include diabetes-associated low-grade inflammation with elevated plasma cytokines, treatment-induced hypoglycemia from insulin or sulfonylureas and compounding factors such as nocturia, advanced age, and comorbidities (39, 40). In addition, elderly patients with T2DM are particularly susceptible due to compounding factors including nocturia, age, and comorbid conditions (41). As a sentinel symptom, excessive sleepiness is suggested to trigger subsequent symptoms such as cognitive and emotional disturbances (41). From a practical life perspective, excessive sleepiness in diabetic patients can significantly impact their self-management abilities, such as diet management and exercise management (42). Meanwhile, studies have also confirmed that this excessive sleepiness may have a certain impact on the quality of life and life expectancy of patients (42). Therefore, mitigating excessive sleepiness may help prevent the onset or progression of other symptoms within this psychological cognition cluster. Therefore, clinical medical staff should take measures to prevent the occurrence of excessive sleepiness, which can also help prevent the onset of other symptoms within the psychological cognition symptom cluster. First, the etiology must be differentiated, specifically ruling out hyperglycemia or hypoglycemia as the causes. Second, glycemic control should be optimized to minimize nocturia-induced sleep disturbances; this involves strict adherence to pharmacotherapy (e.g., insulin or oral hypoglycemic agents) and regular blood glucose monitoring to guide treatment adjustments. Finally, sleep hygiene must be promoted through a regular schedule, an optimized sleep environment, and management of comorbidities like sleep apnea. Clinical staff should document the patients' energy levels, sleep patterns, and timing of drowsiness episodes.

### 4.2 Limb numbness has been identified as the sentinel symptom within the peripheral nerve symptom cluster

This symptom cluster includes limb pain, abnormal sensation in the leg or foot, and soreness in the calf when walking, with limb numbness identified as the sentinel symptom. Studies indicate that approximately 30%−50% of elderly diabetic patients report symmetrical numbness in the distal limbs (e.g., feet, fingers) months to years prior to a formal diagnosis of diabetic neuropathy, with symptoms often progressing to pain, burning, or tingling sensations (43). This observed limb numbness is closely associated with hyperglycemia, which may contribute to axonal degeneration and small-fiber nerve injury (44)_._ It is worth noting that elderly patients often attribute numbness to other chronic conditions (e.g., lumbar degenerative disease, vascular disorders), potentially leading to delayed diagnosis. Furthermore, the incidence of painful diabetic peripheral nerve characterized by spontaneous burning pain or touch-induced pain—increases substantially within 1–3 years following the onset of numbness, can serve as a predictor of painful neuropathy development (45). Therefore, clinical medical staff should recognize the early-warning role of limb numbness and integrate systematic neurological assessments to facilitate the early identification and management of diabetic peripheral nerve. When patients report limb numbness, clinical management should emphasize strict adherence to prescribed hypoglycemic agents or insulin, along with consistent glycemic monitoring to achieve target HbA1c levels, which forms the basis for addressing the underlying pathophysiology. Regular and comprehensive evaluations, including 10-gram monofilament testing and daily foot inspections, are essential to prevent associated complications. Additionally, patients should be educated on the importance of lifestyle interventions, such as smoking cessation, moderate alcohol intake, appropriate physical activity, and balanced nutrition, to help alleviate peripheral neuropathic symptoms.

### 4.3 Limb swelling has been identified as the sentinel symptom within the nephropathy symptom cluster

This symptom cluster includes insomnia, itchy skin, and frothy urine, with limb swelling identified as the sentinel symptom. The underlying mechanism linking limb swelling to other symptoms in this cluster may involve long-term hyperglycemia, which is closely associated with microvascular basement membrane thickening and endothelial dysfunction. In the early stages of diabetic nephropathy, elevated glomerular filtration can lead to sodium and water retention, which may contribute to both limb swelling and the exacerbation of frothy urine (46, 47). Furthermore, resultant limb swelling potentially increases microvascular permeability, facilitating the translocation of inflammatory factors across the blood-brain barrier. This process might inhibit melatonin secretion from the pineal gland, disrupt the sleep-wake cycle, and, thereby, contribute to insomnia (48). Owing to prolonged hyperglycemia, elderly patients with T2DM are particularly susceptible to microvascular dysfunction, which may increase their risk of developing limb edema. Consequently, preventing or managing limb swelling could help mitigate a cascade of symptoms related to renal complications in this population. Therefore, clinical attention should be directed to limb swelling when patients report it. The initial priority is to establish an accurate etiological diagnosis for the patient's edema. Following diagnosis, comprehensive patient education is essential, covering non-pharmacological interventions such as postural adjustments, tailored physical activity, and nutritional management, including adherence to a low-sodium diet (2–3 g/day) (49). Diligent daily monitoring and documentation remain the cornerstone of long-term management, as they are crucial for tracking progression and preventing subsequent related symptoms.

### 4.4 Polydipsia has been identified as the sentinel symptom of the hyperglycemia syndrome symptom cluster

This symptom cluster includes thirst and dry mouth, polyuria, general fatigue and polyuria, with polydipsia identified as the sentinel symptom. The underlying pathogenesis of this cluster appears to be mediated primarily by osmotic diuresis and dehydration resulting from insulin deficiency or resistance (50). A bidirectional relationship is suggested between polyuria and polydipsia, which may further aggravate electrolyte disturbances. Concurrently, persistent hyperglycemia can impair cellular glucose utilization, leading to metabolic dysfunction that often presents clinically as fatigue. Clinical evidence indicates a direct correlation between the intensity of polydipsia and blood glucose levels, with this symptom frequently emerging earlier than other clinical indicators, thereby serving as a practical marker for diabetes screening (51). Collectively, these mechanisms support the view that polydipsia acts as the sentinel symptom of hyperglycemia, which likely contributes to the development of other related symptoms in this cluster. Of clinical relevance is the often-subtle progression of hyperglycemic symptoms in elderly patients with T2DM. Some individuals may present with only mild or intermittent polydipsia, which can be overlooked in routine clinical practice (52). This phenomenon is especially common in elderly patients with T2DM, who tend to attribute such nonspecific symptoms to age-related physiological changes. Therefore, clinical medical staff should maintain a high index of suspicion for hyperglycemia when evaluating patients reporting polydipsia. Measurements of fasting plasma glucose and hemoglobin A1c levels, accompanied by systematic monitoring of fatigue severity as a potential indicator of deteriorating glycemic control.

### 4.5 General fatigue is the core symptom of elderly patients with T2DM

This study differs from that of Liu's research, which may be attributed to the fact that 55.9% of the participants in Liu's study were middle-aged and young adults, whereas the subjects in the present study were all T2DM elderly patients (17). General fatigue is defined as a subjective sense of persistent mental and physical exhaustion (53). It represents the most prevalent symptom among elderly patients with T2DM (54). The pathophysiological basis is primarily attributed to impaired insulin secretion, which reduces ATP synthesis and leads to cellular energy deficiency (55). This metabolic dysfunction is further exacerbated in elderly patients with T2DM by age-related physiological decline and diminished functional capacity. As the core symptom, prolonged unmanaged general fatigue may contribute to the onset of other symptoms and increase the risk of comorbid conditions such as depression (56) and cardiovascular disease (57).

Consequently, clinical medical staff should systematically integrate fatigue assessment into routine clinical monitoring to facilitate early identification and intervention. Evidence-based lifestyle modifications, including traditional Chinese exercises (e.g., Tai Chi) and moderate aerobic activity, should be encouraged (24). Additionally, further investigation into the mechanistic links between fatigue severity and key metabolic parameters—such as glycemic control, inflammatory biomarkers, and nutritional status—is warranted to establish targeted intervention strategies.

## 5 Limitations

First, the study's cross-sectional design limits the ability to establish temporal relationships or track dynamic changes in core and sentinel symptoms among elderly patients with T2DM. Future longitudinal studies are required to elucidate symptom progression patterns over time. Second, as a single-center study conducted at a tertiary hospital in Nanchong, the findings may have limited generalizability. Multicenter investigations involving larger, more diverse samples would improve external validity and support the development of precise, evidence-based symptom management strategies for elderly patients with T2DM in China. Third, symptom assessment in this study may be subject to recall bias. Future research could adopt a prospective longitudinal design, incorporating regular follow-up assessments to record symptom changes dynamically and, thereby, minimize the influence of recall bias on data accuracy. Fourth, the exclusion of low-prevalence symptoms may have affected the analytical findings. Expanding the sample size in future studies would increase the observed frequency of such symptoms, allowing them to be directly included in network analyses and improving the comprehensiveness of the model.

## 6 Conclusion

This study pioneers the integration of symptom network analysis and association rule mining to investigate symptom clusters in elderly patients with T2DM. The analyses identified general fatigue as the core symptom, whereas excessive sleepiness, limb numbness, limb swelling, and polydipsia emerged as sentinel symptoms within their respective clusters. These findings offer new insights into the structure and dynamics of symptom interplay, providing an evidence-based reference for clinical medical staff to develop precise and efficient management strategies aimed at reducing the overall symptom burden in this population.

## 7 Implications for Nursing Practice

(1) Optimize nursing assessment procedures: A dual-focus assessment framework integrating “sentinel symptom early warning” and “core symptom monitoring” should be implemented during routine follow—ups of elderly patients with T2DM. Sentinel symptoms (e.g., excessive daytime sleepiness and limb numbness) should be incorporated into early-risk screening tools, enabling rapid identification of high-risk individuals via brief questionnaires or interviews to anticipate symptom cluster exacerbation. Concurrently, ongoing monitoring of core symptom dynamics—such as general fatigue—is essential to comprehensively track symptom progression.

(2) Develop stratified and personalized intervention strategies: For patients presenting with sentinel symptoms, targeted interventions (e.g., sleep management and psychological support) should be prioritized to disrupt synergistic symptom interactions and prevent cluster deterioration. In patients with prominent core symptoms, symptom-specific care (e.g., fatigue management) should be intensified to maintain the structural stability of symptom clusters and minimize diffuse associations with peripheral symptoms.

(3) Clarify health education priorities: The early-warning significance of sentinel symptoms and the management principles of core symptoms should be communicated to elderly patients and their families. Patients should be guided to actively monitor and promptly report symptom changes, thereby enhancing self-management capacity and establishing a collaborative “clinician-led intervention + patient self-monitoring” model.

## Data Availability

The original contributions presented in the study are included in the article/Supplementary material, further inquiries can be directed to the corresponding author.
