# Supplementary material for: Exploring core and sentinel symptoms in elderly patients with type 2 diabetes through network analysis and the Apriori algorithm
Source: Front Med (Lausanne). 2026 Mar 11;13:1728665. doi: 10.3389/fmed.2026.1728665 (PMC13034604; doi:10.3389/fmed.2026.1728665)
Supplement: Supplementary file 1 [file Data_Sheet_1.docx]

A represents a cluster of psychological cognition symptoms；B represents a cluster of peripheral nerves symptoms；C represents a cluster of gastrointestinal symptoms ；D represents a cluster of hypoglycemia symptom ；E represents a cluster of eye symptoms；F represents a cluster of nephropathy symptoms；G represents a cluster of hyperglycemia symptoms；

A1=Slowed cognition；A2=Asthenia；A3=Excessive sleepiness；A4=Impaired concentration；B1:=Soreness in the calf when walking；B2=Abnormal sensation in the leg or foot；B3=Limb numbness；B4=Limb pain；C1=insomnia；D1=dizziness；D2=Irritable and easily angered；D3=feeling gloomy；E1=Blurred vision；E2=Deterioration of visual acuity；E3=Visual disturbances (flashes or black spots)；F1=Limb swelling；F2=Frothy urine；F3=Insomnia；F4=itchy skin G1=Thirsty and dry mouth；G2=Polyuria；G3=Polydipsia；G4=General fatigue
